# Identifying and measuring the behavioural, dietary, and physical activity components of weight management consultations delivered by general practice nurses in routine care

**DOI:** 10.1186/s12875-021-01403-1

**Published:** 2021-04-07

**Authors:** Heather Tong, Elizabeth Morris, Susan A. Jebb, Dimitrios A. Koutoukidis

**Affiliations:** 1 Nuffield Department of Primary Care Health Sciences, University, of Oxford, Radcliffe Observatory Quarter, Woodstock Road, Oxford, OX2 6GG UK; 2 NIHR Oxford Biomedical Research Centre, Oxford, UK

**Keywords:** Diet, Physical activity, Behaviour change technique, Primary care, Weight loss

## Abstract

**Background:**

Many people with obesity receive weight loss consultations by general practice nurses (GPNs) in routine primary care. This exploratory study aimed to characterise the components of these consultations, including behaviour change techniques (BCTs), and dietary and physical activity recommendations.

**Methods:**

We analysed audio recordings of weight management consultations conducted by 8 GPNs as part of the ‘usual care’ group in a randomised controlled trial (ISRCTN75092026). Consultations were coded against three taxonomies to classify BCTs, dietary recommendations, and physical activity recommendations. Associations between coded content and weight loss were assessed. Differences in the content of consultations where weight loss was < 5% or ≥ 5% from baseline weight at 6 months were explored.

**Results:**

One hundred and fifty audio recordings were available from 53 out of 140 (38%) participants in the usual care group. Participants had on average 3 (SD = 1) recorded consultations over 3 months, lasting 14 (SD = 7) minutes each. Weight change at 3, 6, and 12 months was -3.6% (SD = 4.3), -5.5% (SD = 6.0) and -4.2% (SD = 6.5) for participants with audio recordings. GPNs used 3.9 (SD = 1.6) of 93 BCTs, 3.3 (SD = 2.7) of 30 dietary recommendations and 1.4 (SD = 1.2) of 10 physical activity recommendations per consultation. The most commonly employed BCTs were feedback on outcome of behaviour (80.0%), problem solving (38.0%), and social reward (34.3%). The most common dietary recommendations were about portion size (31.3%), nutrients (28.0%), and balanced diet (19.7%). The main physical activity recommendation was about walking (30.3%). There was no association between weight loss and the number of dietary recommendations, physical activity recommendations, or BCTs used per consultation, or per participant. Social reward was the only technique used significantly more in consultations of participants that lost ≥ 5% of their baseline weight at 6 months.

**Conclusions:**

The study provides a new method that could be used to describe the content of weight management consultations. Specific dietary or physical activity recommendations and BCTs were used infrequently and inconsistently in this group of GPNs. Although replication is required in larger samples, this may point to a weakness in current practice.

**Supplementary Information:**

The online version contains supplementary material available at 10.1186/s12875-021-01403-1.

Contributions to the literature

- This study provides a method to analyse the content of weight management consultations.
- The use of recognised behaviour change techniques or dietary and physical activity recommendations by GPNs was infrequent and variable.
- Results should be generalised with caution, because these GPNs reported training in weight management and self-identified as confident in this area of care

## Background

Obesity affects over 25% of the UK’s population and is a major preventable risk factor for morbidity and mortality [1]. In addition to affecting individuals, obesity places considerable financial strain on healthcare systems and the wider society [2].

Weight loss achieved through changes in diet and physical activity has many benefits including reducing the risk of developing type 2 diabetes, significantly reducing levels of cardiovascular risk factors, such as hypertension, and is associated with a reduction in premature mortality [3–5]. Therefore, weight loss interventions for people with obesity are an important part of wider strategies to reduce non-communicable diseases.

The National Institute for Health and Care Excellence (NICE) recommends that primary care professionals offer behavioural weight management support to people with obesity [6]. One-to-one weight management consultations with a practice GPN are common in the primary care setting. Randomised control trials (RCTs) also frequently use ‘usual care’ interventions as a comparator setting, although the content of these sessions is often neither standardised nor recorded. It is recommended by NICE that weight management consultations should involve discussion of behavioural, dietary, and physical activity change, aiming to achieve a 600 kcal daily energy deficit [6]. This is expected to lead to a 0.5-1 kg weight loss per week [7]. Despite this, most literature finds primary care weight management consultations, including those delivered by practice GPNs, lead to minimal average weight loss, but with substantial variation [8, 9].

Improvements in outcomes may be facilitated by improvements in intervention delivery, but this requires an initial understanding of how GPNs typically conduct weight management consultations [10]. One method of characterising the content of weight management consultations is through the use of taxonomies. Taxonomies enable reporting of complex interventions by identifying ‘active ingredients’: replicable and irreducible components that make an intervention effective. They also establish a common language and can be helpful in translating effective interventions into practice [10]. Characterising the behavioural, diet and physical activity components of GPN-led weight management consultations may allow for the active ingredients of more effective routine consultations to be identified and facilitate intervention optimisation.

This study aimed to address the evidence gap in the content of nurse-led weight management consultations by characterising the behavioural, diet and physical activity components in a small group of consultations. In addition, based on evidence that greater use of behaviour change techniques (BCTs) leads to greater weight loss [11], we aimed to identify whether more intensive use of techniques and recommendations would be associated with weight loss in this study.

## Methods

This was a secondary analysis of data from the Doctor Referral of Overweight People to Low Energy total diet replacement Treatment (DROPLET) trial. The DROPLET trial recruited 278 participants seeking to lose weight and with a BMI ≥ 30 kg/m^2^. Participants were then randomly allocated to a total diet replacement programme or usual care. One hundred and forty participants were allocated to the usual care group, and of this group, 53 participants had their consultations audio-recorded. We report on data from all 53 individuals in the usual care group that had their consultations recorded to avoid introducing further selection bias.

Usual care comprised consultations with a GPN for the explicit purpose of weight loss [12]. The GPNs conducting the consultations had self-identified as confident in delivering a weight management programme. They were asked to continue consultations in their usual manner but additionally received the British Heart Foundation booklet ‘So you want to lose weight for good’ to use at their discretion. Consultations took place in general practices in the UK. Consultations from the trial were audio-recorded, and participants’ weights were recorded at baseline, 3, 6, and 12 months [12].

### Audio recordings

Participants provided written informed consent for their consultations to be recorded. The recordings were not anonymised but were stored securely. MP3 audio recordings of weight management consultations were classified as ‘introductory’ or ‘follow-up’. ‘Introductory’ sessions were the initial meeting between the GPN and each participant, whilst ‘follow-ups’ were subsequent consultations intended to monitor and support the participants. Two audio recordings could not be linked to individual participants, so they were included in descriptive coding but not the analysis of weight change.

### Taxonomy development, use, and coding

Weight management consultations given by GPNs were coded against three components: BCTs using the BCT taxonomy version 1 (BCTTv1) [13], dietary recommendations using a modified version of the Stok 2018 diet taxonomy [14], and physical activity recommendations using an investigator-designed physical activity taxonomy.

The BCTTv1 was chosen as it is an extensive consensually used taxonomy, allowing the identification of BCTs in weight management consultations. The NICE behaviour change guidelines use this taxonomy and include examples of how to use BCTs in the context of weight loss [15]. BCTTv1 contains 93 BCTs grouped into a hierarchy of 16 domains, with examples of a realistic use of each [13]. Three researchers (HT, EM, DAK) coded the content using the established BCTTv1 definitions, available in additional file 1, and examples following completion of a standardised online training for BCTTv1.

The Stok 2018 diet taxonomy was the only diet-specific taxonomy identified within the literature [14]. After listening to randomly selected sample data, the taxonomy was revised to capture the recommendations in the context of weight management consultations more thoroughly. Five recommendations were added: ‘ability to pay’, ‘rate of eating’, ‘food and drink substitution’, ‘total energy intake’, and ‘alcoholic drink intake’. The recommendation ‘dieting’ was removed, so that the specific recommendations used to encourage dieting, a purpose of the consultations, could be quantified. The adapted taxonomy had 30 recommendations within the domains of ‘food choice’, ‘eating behaviour’, or ‘dietary intake and nutrition’. The dietary taxonomy used is available in additional file 2. Researchers coded the content against the adapted taxonomy with pre-set definitions and as examples were not present in the original taxonomy, these were discussed and developed in a code rulebook, available in additional file 3.

A pre-existing physical activity taxonomy could not be identified in the literature, so we developed a taxonomy of 10 recommendations based on the FITT principle (frequency, intensity, time, and type) and a review of existing physical activity interventions [16]. The FITT principle was chosen because it highlights the 4 main planning stages of physical activity. Domains ‘lifestyle activity’ and ‘structured activity’ were chosen as Public Health England (PHE) recognises these as forms of physical activity [17]. The third domain with the single category ‘sedentary time’ was included because sedentary time is independently associated with mortality and morbidity and new PHE guidelines recommend reductions in sedentary time [17, 18]. The physical activity taxonomy used is shown in additional file 4.

Techniques/recommendations were rated as absent (coded as 0), present in all probability (coded as 1), or present beyond all reasonable doubt (coded as 2), as per the BCT training guidance [19]. Coding BCTs/ recommendations as 1 or 2 was based on the confidence of presence, as it was apparent BCTs/ recommendations met the criteria of definitions to differing degrees. One instance of the category present was sufficient to code the content. Consultations were listened to a minimum of 2 times. Coders were blinded to participants’ baseline weight and overall weight change until all coding was complete.

One researcher coded all consultations. Two further researchers independently coded a random sample (10%) of the consultations against the three taxonomies. Coded in batches of two or three, researchers then met to discuss the coding. Justification of the rationale for coding specific BCTs/ recommendations was discussed. Techniques/ recommendations that any researcher coded in consultations that were not coded by both other researchers were discussed in detail. This process was repeated iteratively until an agreement between all 3 coders was reached and final codes were applied.

A code rulebook was developed to aid coding. This consisted of rules on when to code specific BCTs/ recommendations and examples that should and shouldn’t be coded. For example, the BCT social reward was coded as ‘1′ if the GPN gave general reward (e.g. a GPN said well done to a participant, not directly referring to one behaviour in particular but generally from the overall outcome at the end of consultation). It was coded as ‘2′ if the GPN gave reward about a specific behaviour or outcome of behaviour (e.g. if the GPN congratulated the participant for losing 1 kg after weighing them). General coding rules stated to only code what the GPN said. It was not sufficient to code a BCT or recommendation if a GPN was simply responding to a participant’s statement, unless the GPN developed a discussion about the suggestion for an extended period of time. Guidance also stated that when GPNs questioned a participant about their behaviours, it was not sufficient to code questions asking about the past (e.g. ‘Have you swum before?’), but it was sufficient to code if the GPN asked questions relating to the future (e.g. ‘Will you try swimming next week?’).

### Inter-rater reliability

Three random consultations coded for BCTs and 3 for dietary and physical activity recommendations were independently coded by each researcher, following iterative discussion and independent coding in triplicate of 10% of the consultations. Perfect agreement was considered when all coders coded the same values (0, 1 or 2) for a technique/ recommendation. Good and poor agreement, calculated using Krippendorff’s alpha with a SPSS syntax by Hayes, was considered to be an alpha value ≥ 0.600 and < 0.600, respectively.

### Data Analysis

Analyses were carried out in Microsoft Excel v15 and SPSS v25.0 (Chicago, IL). Continuous variables are presented as means and standard deviations. Independent t-tests compared weight change from baseline at 3, 6, and 12 months in audio-recorded and non-audio-recorded groups. The Mann–Whitney U test was used to compare lengths of introductory and follow-up consultations.

Percentage usage of BCTs and recommendations across audio-recorded consultations was calculated. A weighted mean of each BCT and recommendation used in consultations was calculated. BCTs and recommendations coded as 1 were weighted half of those coded as 2, allowing for quantification of the fact that some BCTs were coded as present with more confidence than others.

For the following tests, BCTs and recommendations rated as 1 or 2 were deemed present and calculations were based on presence or absence rather than confidence of presence. Associations between lengths of consultations, and BCT and recommendation use with weight loss were assessed with the Pearson’s correlation coefficient.

Comparisons of the average and total length of consultations as well as the average number of BCTs and recommendations used per consultation were made between participants that had lost at least or less than 5% of their baseline weight at 6 months. This was based on the transtheoretical model of intentional behaviour change which argues long-term changes in behaviour may be assessed after 6 months [20]. Percentage usage of individual BCTs and recommendations used in consultations of the subgroup was compared using chi-squared tests and where appropriate Fisher’s exact test (Table S3, additional file 5).

Due to small numbers, we plotted the weight change and the usage of techniques/ recommendations per consultation by GPN, but were unable to perform additional formal exploratory analysis (Additional file 5, figures S1-4).

The level of statistical significance was set at p < 0.05.

## Results

### Data characteristics

Audio recordings were available for 53 out of 140 participants (38%) in the usual care group of the trial from consultations at 7 practices in Oxfordshire by 8 GPNs. Three additional GPNs delivering consultations did not return any recordings. Participants’ characteristics are detailed in Table 1. Twenty-eight out of 150 audio-recorded consultations (19%) were classified as ‘introductory’ and 122 out of 150 (81%) as ‘follow-up’. Participants in the audio-recorded group had on average 2.8 (SD 1.2) consultations over 3 months, between 1 to 6 in total. The average consultation length per participant was 13.8 (SD 6.7) minutes, ranging from 3.1 to 21.7 min, with no difference in duration between introductory and follow-up consultations (*p* = 0.14). The average total length of all recorded consultations per participant was 36.9 (SD 20.8; range 9.3–82.4) minutes. The British Heart Foundation booklet was discussed in some introductory consultations (20.4%), where it was briefly summarised and given to participants as an extra aide to look through in their own time. It was only discussed rarely thereafter in 4.9% of follow-up consultations.

**Table 1:** Demographic characteristics of participants

Characteristic	All usual care participants (*n* = 140)	Participants with recordings (*n* = 51)	Participants without recordings (*n* = 89)
Age (years)	47.6 (12.7)	49.7 (13.7)	46.2 (11.9)
Sex, female (%)	60.7	64.7	58.4
Ethnicity, white (%)	86.4	82.4	88.7
Baseline weight (kg)	105.1 (19.9)	101.2 (17.7)	107.3 (20.8)

Data presented as mean (SD) or percentage

### Weight change at 3, 6, and 12 months

Weight change in participants with audio-recorded consultations at 3, 6, and 12 months was -3.6% (SD 4.3), -5.5% (SD 6.0), and -4.2% (SD 6.5), respectively. In those without audio recordings, weight change at 3, 6, and 12 months was -3.0% (SD 3.4), -3.8% (SD 5.4), and -2.4% (SD 6.4), respectively. In absolute terms, participants with audio-recorded consultations lost 3.5 kg (SD 4.4), 5.5 kg (SD 6.3), and 4.0 kg (SD 6.7) at 3, 6, and 12 months, respectively, and participants without audio recordings lost 3.1 kg (SD 4.0), 3.8 kg (SD 6.1), and 2.4 kg (SD 6.4), respectively. This modest weight change did not differ significantly between participants with or without audio recordings at 3 months (*p* = 0.37), 6 months (*p* = 0.09), or 12 months (*p* = 0.13).

### Inter-rater reliability

Of the 93 BCTs, 80 were coded with perfect agreement between all coders. A further 2 BCTs had good levels of agreement and 11 BCTs had poor agreement. For dietary recommendations, 25, 1, and 4 recommendations and, for physical activity, 8, 1, and 1 recommendations were coded with perfect, good, and poor agreement, respectively.

### Characterising BCTs, dietary recommendations, and physical activity recommendations used in consultations

**Fig. 1:**
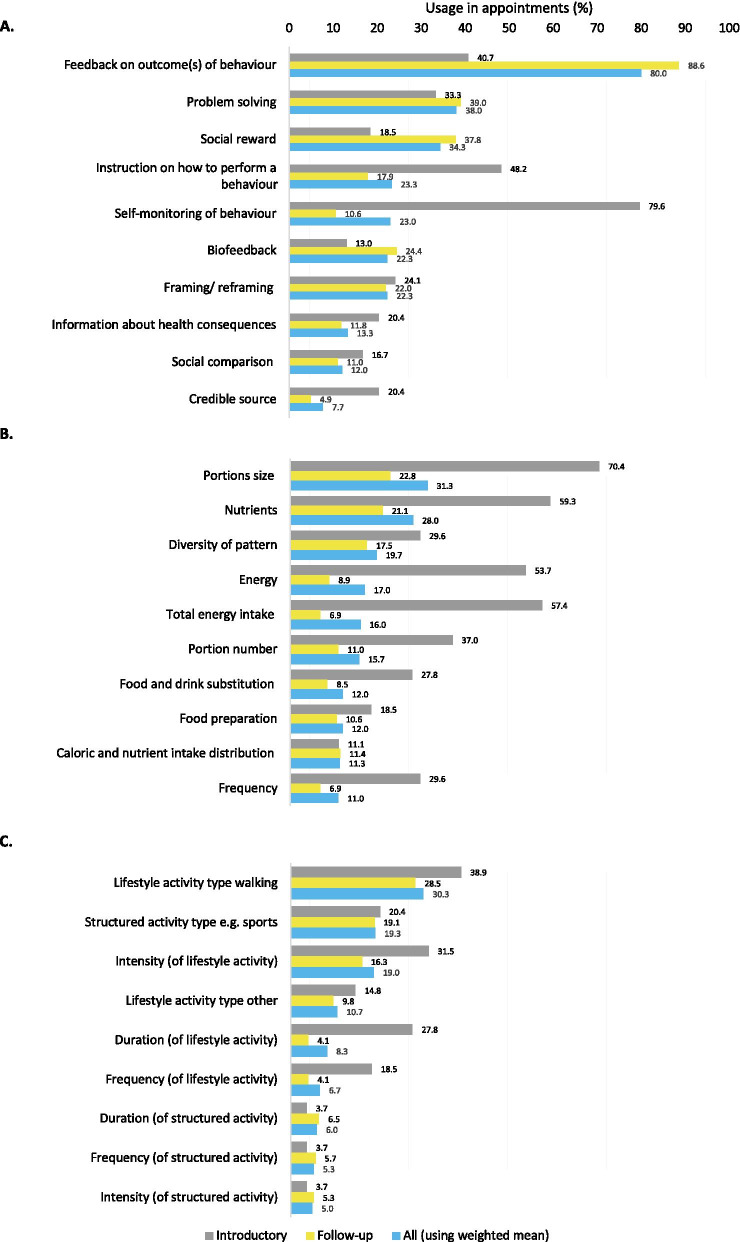
The top ten most frequently used BCTs (**a**), dietary recommendations (**b**) and physical activity recommendations (**c**)

Of 93 possible BCTs, 29 were coded at least once in any consultation from 14 out of 16 domains. Of 30 possible dietary recommendations, 24 were used from all 3 domains and 9 out of 10 physical activity recommendations were used from 2 out of 3 domains (Table 2). The three most frequently used BCTs were ‘feedback on outcome of behaviour’ (used in 80.0% of consultations), ‘problem solving’ (38.0%) and ‘social reward’ (34.3%). The top three dietary recommendations were ‘portion size’ (31.3%), ‘nutrients’ (28.0%), and ‘diversity of pattern’ (19.7%). The most frequent physical activity recommendation was encouragement of walking (30.3%) (Fig. 1).

**Table 2:** Uses of BCTs, dietary and physical activity recommendations across all consultations

Number of	Mean (SD)	Range
BCTs used per consultation per participant	3.9 (1.6)	2–9
BCTs used per participant over all of a participant’s consultations	10.6 (5.4)	3–22
Dietary recommendations used per consultation per participant	3.3 (2.7)	0–12
Dietary recommendations used over all of a participant’s consultations	8.4 (6.6)	0–27
Physical activity recommendations used per consultation per participant	1.4 (1.2)	0–5
Physical activity recommendations used over all of a participant’s consultations	3.6 (3.5)	0–11

### Associations between consultations, components and outcomes

The length of consultations was significantly positively associated with the number of BCTs present (*r* = 0.45, *p* = 0.01). The length of consultation was not associated with the number of dietary (*r* = 0.28, *p* = 0.16) or physical activity recommendations (*r* = 0.04, *p* = 0.83). The total length of all audio recordings a participant received was not associated with weight change at 3 (*r* = -0.03, *p* = 0.84), 6 (*r* = 0.01, *p* = 0.93), or 12 (*r* = -0.05, *p* = 0.78) months.

There were no significant associations between the total number of BCTs, dietary or physical activity recommendations used in participants’ introductory consultations with weight change at 3, 6, or 12 months or the total number used across all of a participant’s consultations and weight change at 3, 6 or 12 months (Table 3). There were also no significant correlations between the average numbers of BCTs, dietary or physical activity recommendations used per consultation with weight change at 3, 6, or 12 months, with the exception of an association between the average number of physical activity recommendations used per consultation and weight gain at 12 months only (r = 0.36 (*p* = 0.02), Tables S1-S3, additional file 5).

**Table 3:** Correlation coefficients (*p*-values) between the total number of BCTs or recommendations used in introductory and all consultations of participants and percentage weight change at 3, 6 and 12 months

		Total numbers of BCTs coded	Total number of dietary recommendations coded	Total number of physical activity recommendations coded
3-month percentage weight change	Introductory (*n* = 21)	0.21 (0.36)	-0.07 (0.76)	0.10 (0.65)
	All consultations (*n* = 40)	-0.21 (0.19)	-0.11 (0.50)	0.20 (0.22)
6-month percentage weight change	Introductory (*n* = 20)	0.17 (0.49)	-0.28 (0.24)	0.13 (0.44)
	All consultations (*n* = 39)	-0.06 (0.72)	-0.14 (0.38)	0.23 (0.16)
12-month percentage weight change	Introductory (*n* = 20)	0.18 (0.44)	-0.22 (0.35)	0.34 (0.14)
	All consultations (*n* = 40)	0.00 (1.00)	-0.03 (0.87)	0.29 (0.07)

A negative correlation indicated that the number of techniques / recommendations was positively associated with weight loss

On visual inspection of figures S1-4 (additional file 5) we found no evidence of differences in participant weight change or use of BCTs and recommendations by the GPN delivering the intervention.

### Comparison of BCT, dietary and physical activity recommendation use by weight loss of participants less or at least 5% from baseline at 6 months

Thirty-nine (74%) participants with audio recordings had 6-month weight data. Nineteen participants (49%) had lost ≥ 5% of their baseline weight at 6 months and 59 consultations were recorded. The mean number and length of consultations per participant was 3.11 (SD 1.2) and 13.6 (SD 5.0) minutes respectively. In the 20 participants that lost < 5% of their weight (51%), there were 62 recorded consultations. Mean number and length of consultations per participant was 3.15 (SD 1.0) and 12.6 (SD 4.8) minutes respectively. There were no significant differences in the mean length of consultations (*p* = 0.84) or total length of all audio-recorded consultations (*p* = 0.26) between the groups.

There were no significant differences between the two groups in the number of BCTs, dietary or physical activity recommendations used per consultation with the exception of social reward which was used significantly more in the group that lost ≥ 5% of their baseline weight. The BCTs and recommendations used in consultations and their comparisons are available in additional file 5, tables S2 and S3.

## Discussion

Characterising the use of BCTs, dietary recommendations, and physical activity recommendations in this small group of GPN-led weight management consultations showed their use to be infrequent and variable. Despite consultations lasting longer (13.8 min) than typical primary care consultations (9.2 min) [21], few recognised BCTs, dietary or physical activity recommendations were used. Only 31% of BCTs were used and although 80% of the possible dietary recommendations were used, only 3/30 recommendations were used per consultation. Only 1/10 physical activity recommendation was used per consultation. GPNs used less than one BCT or recommendation per minute of consultation, and the number of BCTs or recommendations used were not significantly associated with weight loss.

This study has several strengths. Three different taxonomies were used to comprehensively characterise the relevant consultation components. The BCT taxonomy is well established, and the existing dietary taxonomy was adapted to improve characterisation. The novel physical activity taxonomy was developed based on an existing framework, and for the first time allowed coding of physical activity recommendations. GPNs were encouraged to carry out consultations as they had routinely done in their practice without the use of a prescriptive protocol, or additional training, allowing a more realistic characterisation of consultations in everyday clinical practice. Consultations were coded blind to outcome and inter-rater agreement levels were good. Although this is a specific and selected group of GPNs leading weight management consultations, this study indicates that what happens in "usual care” may or may not be grounded in guidelines. In the future, analysis of the content of usual care groups, potentially with the methods proposed here, and more standardised reporting, could help to identify effective components and improve reproducibility.

The study also has some limitations. About a third of consultations were recorded, which may reflect reluctance on the part of the GPN to record their consultations. There were no significant differences between weight change in the groups with and without audio recordings, but it is not possible to know whether the content of the recorded consultations are representative of the whole usual care group. Out of 11 GPNs, 8 GPNs submitted recordings and data were unavailable regarding the number or length of consultations participants without audio recordings received. There was no information on whether any additional consultations occurred outside of the recorded consultations, but we consider it unlikely that a significant number of additional consultations would exist, since this is neither routine care nor was advised in the trial. Overall, the small sample size limits the generalisability of our findings. The amount or type of training each GPN had on weight management was not recorded, but we found no evidence of differences in the components of the consultations or participants’ weight loss between GPNs. Lack of formal analysis of the appropriateness and individualisation of recommendation use is also a limitation. Multiple testing might have led to increased probability for type I error, which limits our confidence in the few significant correlations identified. The observational design does not allow for casual inferences.

Previous research has found that primary care staff feel ill-equipped to help people with obesity, often reporting that they have inadequate training [22]. GPNs in this group, with previous training in weight management used only 46% of possible techniques/ recommendations on one occasion, and only 17% were used in more than 10% of consultations. Though it would not be expected for GPNs to use all BCTs and recommendations in one consultation, this low usage may reflect lack of awareness of effective weight-loss strategies. The literature suggests that increased and more adherent usage of BCTs is associated with greater weight loss [11], and the low and infrequent use of BCTs might explain the lack of association with weight loss shown here. Direct comparisons with other studies are difficult regarding dietary and physical activity recommendations as this is the first study using the taxonomies developed, but the lack of correlation between the frequency of dietary and physical activity recommendations given in consultations and weight loss may also result from low levels of use.

GPNs’ use of techniques/ recommendations was confined to a small group of specific BCTs and recommendations. The most frequently used BCT was ‘feedback on outcome of behaviour’, which has been linked to improved weight loss outcomes [23]. Reviews of weight management consultations commonly report ‘self-monitoring of behaviour’, used in almost 80% of introductory consultations, as one of the most effective BCTs to promote weight loss too [24, 25] and participants’ weight loss, though modest, was better than other UK GPN led BMWCs [8, 26]. ‘Social reward’ was the third most commonly used BCT and used significantly more in the group that lost ≥ 5% baseline weight at 6 months. NICE recommends healthcare professionals should ‘praise successes’ when offering weight management support [6] and it may be that as patients lost weight, GPNs congratulated patients. Additionally, it has been found that positive reinforcement of behaviour can be effective in other health behaviours, such as in increasing physical activity levels [27]. In terms of specific dietary recommendations, reference to ‘energy’ and ‘total energy intake’ were used in just over 15% of consultations characterised. Energy restriction is known to be a key component for weight loss [28, 29]. However, there was very limited discussion of specific energy deficits in the consultations and the lack of use in association with the BCT ‘goal setting’ could have limited weight loss here.

## Conclusions

Specific dietary or physical activity recommendations and BCTs were used infrequently and variably in this small group of GPN-led weight management consultations. Further analysis in larger and representative samples is warranted. Future studies could usefully examine if additional training can increase the quality and effectiveness of consultations.

## Supplementary Information

**Additional file 1.** BCTs, their descriptions and examples provided by BCTTv1 used by coders. This taxonomy has been taken directly from Michie et al’s paper for reference.

**Additional file 2.** Dietary recommendations and their definitions used by coders. This has been adapted after taking directly from Stok et Al’s paper.

**Additional file 3. **Coding rulebook.

**Additional file 4.** Physical activity recommendations and their definitions used by coders.

**Additional file 5. **Supplementary results.

## Data Availability

The data are not publicly available due to them containing information that could compromise research participant privacy. Individual participant data that are deidentified and support the findings of this study are available from the chief investigator (SAJ) upon reasonable request. Requests for access to data should be sent to susan.jebb@phc.ox.ac.uk. All proposals requesting data access will need to complete a data request form with details of the research question and analysis plan. All proposals will require the approval of the investigator team before any data are released.

## Abbreviations

BCT: Behaviour change technique
DROPLET: Doctor referral of overweight people to a low-energy treatment
GPN: General practice nurse
PHE: Public Health England
NICE: National Institute for Health and Care Excellence
RCT: Randomised control trial
