# Supplementary material for: Identifying and measuring the behavioural, dietary, and physical activity components of weight management consultations delivered by general practice nurses in routine care
Source: BMC Fam Pract. 2021 Apr 7;22:65. doi: 10.1186/s12875-021-01403-1 (PMC8028812; doi:10.1186/s12875-021-01403-1)
Supplement: Supplementary file 1 — Additional file 1. BCTs, their descriptions and examples provided by BCTTv1 used by coders. This taxonomy has been taken directly from Michie et al’s paper for reference. [file 12875_2021_1403_MOESM1_ESM.pdf]

Additional file 1. BCTs, their descriptions and examples provided by BCTTv1 used by coders. This taxonomy has been taken directly from Michie et al's paper for reference.

BCT Reference	Name	Description	Example
#1.1	Goal setting (behaviour)	Set or agree on a goal defined in terms of the behaviour to be achieved	Agree on a daily walking goal (e.g. 3 miles) with the person and reach agreement about the goal<br>Set the goal of eating 5 pieces of fruit per day as specified in public health guidelines
#1.2	Problem solving	Analyse, or prompt the person to analyse, factors influencing the behaviour and generate or select strategies that include overcoming barriers and/or increasing facilitators (includes 'Relapse Prevention' and 'Coping Planning')	Identify specific triggers (e.g. being in a pub, feeling anxious) that generate the urge/want/need to drink and develop strategies for avoiding environmental triggers or for managing negative emotions, such as anxiety, that motivate drinking
#1.3	Goal setting (outcome)	Set or agree on a goal defined in terms of a positive outcome of wanted behaviour	Set a weight loss goal (e.g. 0.5 kilogram over one week) as an outcome of changed eating patterns
#1.4	Action planning	Prompt detailed planning of performance of the behaviour (must include at least one of context, frequency, duration and intensity). Context may be environmental (physical or social) or internal (physical, emotional or cognitive) (includes 'Implementation Intentions')	Encourage a plan to carry condoms when going out socially at weekends<br><br>Prompt planning the performance of a particular physical activity (e.g. running) at a particular time (e.g. before work) on certain days of the week
#1.5	Review behaviour goal(s)	Review behaviour goal(s) jointly with the person and consider modifying goal(s) or behaviour change strategy in light of achievement. This may lead to re-setting the same goal, a small change in that goal or setting a new goal instead of (or in addition to) the first, or no change	Examine how well a person's performance corresponds to agreed goals e.g. whether they consumed less than one unit of alcohol per day, and consider modifying future behavioural goals accordingly e.g. by increasing or decreasing alcohol target or changing type of alcohol consumed
#1.6	Discrepancy between current behaviour and goal	Draw attention to discrepancies between a person's current behaviour (in terms of the form, frequency, duration, or intensity of that behaviour) and the person's previously set outcome goals, behavioural goals or action plans (goes beyond self-monitoring of behaviour)	Point out that the recorded exercise fell short of the goal set
#1.7	Review outcome goal(s)	Review outcome goal(s) jointly with the person and consider modifying goal(s) in light of achievement. This may lead to re-setting the same goal, a small change in that goal or setting a new goal instead of, or in addition to the first	Examine how much weight has been lost and consider modifying outcome goal(s) accordingly e.g., by increasing or decreasing subsequent weight loss targets
#1.8	Behavioural contract	Create a written specification of the behaviour to be performed, agreed on by the person, and witnessed by another	Sign a contract with the person e.g. specifying that they will not drink alcohol for one week
#1.9	Commitment	Note: if defined in terms of the behaviour to be achieved also code 1.1, Goal setting (behaviour)	Ask the person to use an 'I will' statement to affirm or reaffirm a strong commitment (i.e. using the words 'strongly', 'committed' or 'high priority') to start, continue or restart the attempt to take medication as prescribed

#2.1	Monitoring of behaviour by others without feedback	Observe or record behaviour with the person's knowledge as part of a behaviour change strategy	Watch hand washing behaviours among health care staff and make notes on context, frequency and technique used
#2.2	Feedback on behaviour	Monitor and provide informative or evaluative feedback on performance of the behaviour (e.g. form, frequency, duration, intensity)	Inform the person of how many steps they walked each day (as recorded on a pedometer) or how many calories they ate each day (based on a food consumption questionnaire).
#2.3	Self-monitoring of behaviour	Establish a method for the person to monitor and record their behaviour(s) as part of a behaviour change strategy	Ask the person to record daily, in a diary, whether they have brushed their teeth for at least two minutes before going to bed. Give patient a pedometer and a form for recording daily total number of steps.
#2.4	Self-monitoring of outcome(s) of behaviour	Establish a method for the person to monitor and record the outcome(s) of their behaviour as part of a behaviour change strategy	Ask the person to weigh themselves at the end of each day, over a two week period, and record their daily weight on a graph to increase exercise behaviours
#2.5	Monitoring outcome(s) of behaviour by others without feedback	Observe or record outcomes of behaviour with the person's knowledge as part of a behaviour change strategy	Record blood pressure, blood glucose, weight loss, or physical fitness
#2.6	Biofeedback	Provide feedback about the body (e.g. physiological or biochemical state) using an external monitoring device as part of a behaviour change strategy	Inform the person of their blood pressure reading to improve adoption of health behaviours
#2.7	Feedback on outcome(s) of behaviour	Monitor and provide feedback on the outcome of performance of the behaviour	Inform the person of how much weight they have lost following the implementation of a new exercise regime
#3.1	Social support (unspecified)	Advise on, arrange or provide social support (e.g. from friends, relatives, colleagues,' buddies' or staff) or non-contingent praise or reward for performance of the behaviour. It includes encouragement and counselling, but only when it is directed at the behaviour	Advise the person to call a 'buddy' when they experience an urge to smoke. Arrange for a housemate to encourage continuation with the behaviour change programme. Give information about a self-help group that offers support for the behaviour
#3.2	Social support (practical)	Advise on, arrange, or provide practical help (e.g. from friends, relatives, colleagues, 'buddies' or staff) for performance of the behaviour	Ask the partner of the patient to put their tablet on the breakfast tray so that the patient remembers to take it
#3.3	Social support (emotional)	Advise on, arrange, or provide emotional social support (e.g. from friends, relatives, colleagues, 'buddies' or staff) for performance of the behaviour	Ask the patient to take a partner or friend with them to their colonoscopy appointment
#4.1	Instruction on how to perform a behaviour	Advise or agree on how to perform the behaviour (includes 'Skills training')	Advise the person how to put a condom on a model of a penis correctly
#4.2	Information about antecedents	Provide information about antecedents (e.g. social and environmental situations and events, emotions, cognitions) that reliably predict performance of the behaviour	Advise to keep a record of snacking and of situations or events occurring prior to snacking
#4.3	Re-attribution	Elicit perceived causes of behaviour and suggest alternative explanations (e.g.	If the person attributes their over-eating to the frequent presence of delicious food, suggest that the 'real' cause may be the

		external or internal and stable or unstable)	person's inattention to bodily signals of hunger and satiety
#4.4	Behavioural experiments	Advise on how to identify and test hypotheses about the behaviour, its causes and consequences, by collecting and interpreting data	Ask a family physician to give evidence-based advice rather than prescribe antibiotics and to note whether the patients are grateful or annoyed
#5.1	Information about health consequence s	Provide information (e.g. written, verbal, visual) about health consequences of performing the behaviour	Explain that not finishing a course of antibiotics can increase susceptibility to future infection<br>Present the likelihood of contracting a sexually transmitted infection following unprotected sexual behaviour
#5.2	Salience of consequence s	Use methods specifically designed to emphasise the consequences of performing the behaviour with the aim of making them more memorable (goes beyond informing about consequences)	Produce cigarette packets showing pictures of health consequences e.g. diseased lungs, to highlight the dangers of continuing to smoke
#5.3	Information about social and environment al consequence s	Provide information (e.g. written, verbal, visual) about social and environmental consequences of performing the behaviour	Tell family physician about financial remuneration for conducting health screening<br>Inform a smoker that the majority of people disapprove of smoking in public places
#5.4	Monitoring of emotional consequence s	Prompt assessment of feelings after attempts at performing the behaviour	Agree that the person will record how they feel after taking their daily walk
#5.5	Anticipated regret	Induce or raise awareness of expectations of future regret about performance of the unwanted behaviour	Ask the person to assess the degree of regret they will feel if they do not quit smoking
#5.6	Information about emotional consequence s	Provide information (e.g. written, verbal, visual) about emotional consequences of performing the behaviour	Explain that quitting smoking increases happiness and life satisfaction
#6.1	Demonstratio n of the behaviour	Provide an observable sample of the performance of the behaviour, directly in person or indirectly e.g. via film, pictures, for the person to aspire to or imitate (includes 'Modelling').	Demonstrate to nurses how to raise the issue of excessive drinking with patients via a role-play exercise
#6.2	Social comparison	Draw attention to others' performance to allow comparison with the person's own performance Note: being in a group setting does not necessarily mean that social comparison is actually taking place	Show the doctor the proportion of patients who were prescribed antibiotics for a common cold by other doctors and compare with their own data
#6.3	Information about others' approval	Provide information about what other people think about the behaviour. The information clarifies whether others will like, approve or disapprove of what the person is doing or will do	Tell the staff at the hospital ward that staff at all other wards approve of washing their hands according to the guidelines
#7.1	Prompts/cue s	Introduce or define environmental or social stimulus with the purpose of prompting or cueing the behaviour. The prompt or cue would normally occur at the time or place of performance	Put a sticker on the bathroom mirror to remind people to brush their teeth
#7.2	Cue signalling reward	Identify an environmental stimulus that reliably predicts that reward will follow the behaviour (includes 'Discriminative cue')	Advise that a fee will be paid to dentists for a particular dental treatment of 6-8 year old, but not older, children to encourage

			delivery of that treatment (the 6-8 year old children are the environmental stimulus)
#7.3	Reduce prompts/cues	Withdraw gradually prompts to perform the behaviour (includes 'Fading')	Reduce gradually the number of reminders used to take medication
#7.4	Remove access to the reward	Advise or arrange for the person to be separated from situations in which unwanted behaviour can be rewarded in order to reduce the behaviour (includes 'Time out')	Arrange for cupboard containing high calorie snacks to be locked for a specified period to reduce the consumption of sugary foods in between meals
#7.5	Remove aversive stimulus	Advise or arrange for the removal of an aversive stimulus to facilitate behaviour change (includes 'Escape learning')	Arrange for a gym-buddy to stop nagging the person to do more exercise in order to increase the desired exercise behaviour
#7.6	Satiation	Advise or arrange repeated exposure to a stimulus that reduces or extinguishes a drive for the unwanted behaviour	Arrange for the person to eat large quantities of chocolate, in order to reduce the person's appetite for sweet foods
#7.7	Exposure	Provide systematic confrontation with a feared stimulus to reduce the response to a later encounter	Agree a schedule by which the person who is frightened of surgery will visit the hospital where they are scheduled to have surgery
#7.8	Associative learning	Present a neutral stimulus jointly with a stimulus that already elicits the behaviour repeatedly until the neutral stimulus elicits that behaviour (includes 'Classical/Pavlovian Conditioning')	Present repeatedly fatty foods with a disliked sauce to discourage the consumption of fatty foods
#8.1	Behavioural practice/ rehearsal	Prompt practice or rehearsal of the performance of the behaviour one or more times in a context or at a time when the performance may or may not be necessary, in order to increase habit and skill	Prompt asthma patients to practice measuring their peak flow in the nurse's consulting room
#8.2	Behaviour substitution	Prompt substitution of the unwanted behaviour with a wanted or neutral behaviour	Suggest that the person goes for a walk rather than watches television
#8.3	Habit formation	Prompt rehearsal and repetition of the behaviour in the same context repeatedly so that the context elicits the behaviour	Prompt patients to take their statin tablet before brushing their teeth every evening
#8.4	Habit reversal	Prompt rehearsal and repetition of an alternative behaviour to replace an unwanted habitual behaviour	Ask the person to walk up stairs at work where they previously always took the lift
#8.5	Overcorrection	Ask to repeat the wanted behaviour in an exaggerated way following an unwanted behaviour	Ask to eat only fruit and vegetables the day after a poor diet
#8.6	Generalisation of a target behaviour	Advise to perform the wanted behaviour, which is already performed in a particular situation, in another situation	Advise to repeat toning exercises learned in the gym when at home
#8.7	Graded tasks	Set easy-to-perform tasks, making them increasingly difficult, but achievable, until behaviour is performed	Ask the person to walk for 100 yards a day for the first week, then half a mile a day after they have successfully achieved 100 yards, then two miles a day after they have successfully achieved one mile
#9.1	Credible source	Present verbal or visual communication from a credible source in favour of or against the behaviour	Present a speech given by a high status professional to emphasise the importance of not exposing patients to unnecessary radiation by ordering x-rays for back pain
#9.2	Pros and cons	Advise the person to identify and compare reasons for wanting (pros) and not wanting to (cons) change the behaviour (includes 'Decisional balance')	Advise the person to list and compare the advantages and disadvantages of prescribing antibiotics for upper respiratory tract infections

#9.3	Comparative imagining of future outcomes	Prompt or advise the imagining and comparing of future outcomes of changed versus unchanged behaviour	Prompt the person to imagine and compare likely or possible outcomes following attending versus not attending a screening appointment
#10.1	Material incentive (behaviour)	Inform that money, vouchers or other valued objects will be delivered if and only if there has been effort and/or progress in performing the behaviour (includes 'Positive reinforcement')	Inform that a financial payment will be made each month in pregnancy that the woman has not smoked
#10.2	Material reward (behaviour)	Arrange for the delivery of money, vouchers or other valued objects if and only if there has been effort and/or progress in performing the behaviour (includes 'Positive reinforcement')	Arrange for the person to receive money that would have been spent on cigarettes if and only if the smoker has not smoked for one month
#10.3	Non-specific reward	Arrange delivery of a reward if and only if there has been effort and/or progress in performing the behaviour (includes 'Positive reinforcement')	Identify something (e.g. an activity such as a visit to the cinema) that the person values and arrange for this to be delivered if and only if they attend for health screening
#10.4	Social reward	Arrange verbal or non-verbal reward if and only if there has been effort and/or progress in performing the behaviour (includes 'Positive reinforcement')	Congratulate the person for each day they eat a reduced fat diet
#10.5	Social incentive	Inform that a verbal or non-verbal reward will be delivered if and only if there has been effort and/or progress in performing the behaviour (includes 'Positive reinforcement')	Inform that they will be congratulated for each day they eat a reduced fat diet
#10.6	Non-specific incentive	Inform that a reward will be delivered if and only if there has been effort and/or progress in performing the behaviour (includes 'Positive reinforcement')	Identify an activity that the person values and inform them that this will happen if and only if they attend for health screening
#10.7	Self-incentive	Plan to reward self in future if and only if there has been effort and/or progress in performing the behaviour	Encourage to provide self with material (e.g., new clothes) or other valued objects if and only if they have adhered to a healthy diet
#10.8	Incentive (outcome)	Inform that a reward will be delivered if and only if there has been effort and/or progress in achieving the behavioural outcome (includes 'Positive reinforcement')	Inform the person that they will receive money if and only if a certain amount of weight is lost
#10.9	Self-reward	Prompt self-praise or self-reward if and only if there has been effort and/or progress in performing the behaviour	Encourage to reward self with material (e.g., new clothes) or other valued objects if and only if they have adhered to a healthy diet
#10.10	Reward (outcome)	Arrange for the delivery of a reward if and only if there has been effort and/or progress in achieving the behavioural outcome (includes 'Positive reinforcement')	Arrange for the person to receive money if and only if a certain amount of weight is lost
#10.11	Future punishment	Inform that future punishment or removal of reward will be a consequence of performance of an unwanted behaviour (may include fear arousal) (includes 'Threat')	Inform that continuing to consume 30 units of alcohol per day is likely to result in loss of employment if the person continues
#11.1	Pharmacological support	Provide, or encourage the use of or adherence to, drugs to facilitate behaviour change	Suggest the patient asks the family physician for nicotine replacement therapy to facilitate smoking cessation
#11.2	Reduce negative emotions	Advise on ways of reducing negative emotions to facilitate performance of the behaviour (includes 'Stress Management')	Advise on the use of stress management skills, e.g. to reduce anxiety about joining Alcoholics Anonymous

#11.3	Conserving mental resources	Advise on ways of minimising demands on mental resources to facilitate behaviour change	Advise to carry food calorie content information to reduce the burden on memory in making food choices
#11.4	Paradoxical instructions	Advise to engage in some form of the unwanted behaviour with the aim of reducing motivation to engage in that behaviour	Advise a smoker to smoke twice as many cigarettes a day as they usually do<br>Tell the person to stay awake as long as possible in order to reduce insomnia
#12.1	Restructuring the physical environment	Change, or advise to change the physical environment in order to facilitate performance of the wanted behaviour or create barriers to the unwanted behaviour (other than prompts/cues, rewards and punishments)	Advise to keep biscuits and snacks in a cupboard that is inconvenient to get to<br>Arrange to move vending machine out of the school
#12.2	Restructuring the social environment	Change, or advise to change the social environment in order to facilitate performance of the wanted behaviour or create barriers to the unwanted behaviour (other than prompts/cues, rewards and punishments)	Advise to minimise time spent with friends who drink heavily to reduce alcohol consumption
#12.3	Avoidance/reducing exposure to cues for the behaviour	Advise on how to avoid exposure to specific social and contextual/physical cues for the behaviour, including changing daily or weekly routines	Suggest to a person who wants to quit smoking that their social life focus on activities other than pubs and bars which have been associated with smoking
#12.4	Distraction	Advise or arrange to use an alternative focus for attention to avoid triggers for unwanted behaviour	Suggest to a person who is trying to avoid between-meal snacking to focus on a topic they enjoy (e.g. holiday plans) instead of focusing on food
#12.5	Adding objects to the environment	Add objects to the environment in order to facilitate performance of the behaviour	Provide free condoms to facilitate safe sex<br>Provide attractive toothbrush to improve tooth brushing technique
#12.6	Body changes	Alter body structure, functioning or support directly to facilitate behaviour change	Prompt strength training, relaxation training or provide assistive aids (e.g. a hearing aid)
#13.1	Identification of self as role model	Inform that one's own behaviour may be an example to others	Inform the person that if they eat healthily, that may be a good example for their children
#13.2	Framing/ reframing	Suggest the deliberate adoption of a perspective or new perspective on behaviour (e.g. its purpose) in order to change cognitions or emotions about performing the behaviour (includes 'Cognitive structuring')	Suggest that the person might think of the tasks as reducing sedentary behaviour (rather than increasing activity)
#13.3	Incompatible beliefs	Draw attention to discrepancies between current or past behaviour and self-image, in order to create discomfort (includes 'Cognitive dissonance')	Draw attention to a doctor's liberal use of blood transfusion and their self-identification as a proponent of evidence-based medical practice
#13.4	Valued self-identity	Advise the person to write or complete rating scales about a cherished value or personal strength as a means of affirming the person's identity as part of a behaviour change strategy (includes 'Self-affirmation')	Advise the person to write about their personal strengths before they receive a message advocating the behaviour change
#13.5	Identity associated with changed behaviour	Advise the person to construct a new self-identity as someone who 'used to engage with the unwanted behaviour'	Ask the person to articulate their new identity as an 'ex-smoker'
#14.1	Behaviour cost	Arrange for withdrawal of something valued if and only if an unwanted behaviour is performed (includes 'Response cost').	Subtract money from a prepaid refundable deposit when a cigarette is smoked

#14.2	Punishment	Arrange for aversive consequence contingent on the performance of the unwanted behaviour	Arrange for the person to wear unattractive clothes following consumption of fatty foods
#14.3	Remove reward	Arrange for discontinuation of contingent reward following performance of the unwanted behaviour (includes 'Extinction')	Arrange for the other people in the household to ignore the person every time they eat chocolate (rather than attending to them by criticising or persuading)
#14.4	Reward approximation	Arrange for reward following any approximation to the target behaviour, gradually rewarding only performance closer to the wanted behaviour (includes 'Shaping')	Arrange reward for any reduction in daily calories, gradually requiring the daily calorie count to become closer to the planned calorie intake
#14.5	Rewarding completion	Build up behaviour by arranging reward following final component of the behaviour; gradually add the components of the behaviour that occur earlier in the behavioural sequence (includes 'Backward chaining')	Reward eating a supplied low calorie meal; then make reward contingent on cooking and eating the meal; then make reward contingent on purchasing, cooking and eating the meal
#14.6	Situation-specific reward	Arrange for reward following the behaviour in one situation but not in another (includes 'Discrimination training')	Arrange reward for eating at mealtimes but not between meals
#14.7	Reward incompatible behaviour	Arrange reward for responding in a manner that is incompatible with a previous response to that situation (includes 'Counter-conditioning')	Arrange reward for ordering a soft drink at the bar rather than an alcoholic beverage
#14.8	Reward alternative behaviour	Arrange reward for performance of an alternative to the unwanted behaviour (includes 'Differential reinforcement')	Reward for consumption of low fat foods but not consumption of high fat foods
#14.9	Reduce reward frequency	Arrange for rewards to be made contingent on increasing duration or frequency of the behaviour (includes 'Thinning')	Arrange reward for each day without smoking, then each week, then each month, then every 2 months and so on
#14.10	Remove punishment	Arrange for removal of an unpleasant consequence contingent on performance of the wanted behaviour (includes 'Negative reinforcement')	Arrange for someone else to do housecleaning only if the person has adhered to the medication regimen for a week
#15.1	Verbal persuasion about capability	Tell the person that they can successfully perform the wanted behaviour, arguing against self-doubts and asserting that they can and will succeed	Tell the person that they can successfully increase their physical activity, despite their recent heart attack.
#15.2	Mental rehearsal of successful performance	Advise to practise imagining performing the behaviour successfully in relevant contexts	Advise to imagine eating and enjoying a salad in a work canteen
#15.3	Focus on past success	Advise to think about or list previous successes in performing the behaviour (or parts of it)	Advise to describe or list the occasions on which the person had ordered a non-alcoholic drink in a bar
#15.4	Self-talk	Prompt positive self-talk (aloud or silently) before and during the behaviour	Prompt the person to tell themselves that a walk will be energising
#16.1	Imaginary punishment	Advise to imagine performing the unwanted behaviour in a real-life situation followed by imagining an unpleasant consequence (includes 'Covert sensitisation')	Advise to imagine overeating and then vomiting
#16.2	Imaginary reward	Advise to imagine performing the wanted behaviour in a real-life situation followed by imagining a pleasant consequence (includes 'Covert conditioning')	Advise the health professional to imagine giving dietary advice followed by the patient losing weight and no longer being diabetic

#16.3	Vicarious consequences	Prompt observation of the consequences (including rewards and punishments) for others when they perform the behaviour	Draw attention to the positive comments other staff get when they disinfect their hands regularly
