# Supplementary material for: Identifying and measuring the behavioural, dietary, and physical activity components of weight management consultations delivered by general practice nurses in routine care
Source: BMC Fam Pract. 2021 Apr 7;22:65. doi: 10.1186/s12875-021-01403-1 (PMC8028812; doi:10.1186/s12875-021-01403-1)
Supplement: Supplementary file 2 — Additional file 2. Dietary recommendations and their definitions used by coders. This has been adapted after taking directly from Stok et Al’s paper. [file 12875_2021_1403_MOESM2_ESM.pdf]

Additional file 2. Dietary recommendations and their definitions used by coders. This has been adapted after taking directly from Stok et Al's paper.

Reference	Name	Definition
1.1	Preferences	The extent to which food items are considered desirable or undesirable including underlying preferences (attitudes toward, e.g., taste, odor, color, or quality and health-related concerns) and expressed preferences (actual choice of one food item over another) (Rozin, 2006; Bernheim and Rangel, 2009).
1.2	Ability to pay	The extent to which one is able to buy certain food products
1.3	Share of income spent on food	The share of income (of one person, family, or household) that is spent on the totality of food one consumes (Clements and Si, 2017).
1.4	Willingness-to-pay	Largest sum of money one is willing to pay for a certain food product (Varian, 1992).
1.5	Frequency of purchase	How often a certain food product is purchased in a specific time frame (Kim and Rossi, 1994).
1.6	Product of purchase	Which food product is purchased (Mohd Suki, 2016). Influenced by looking at food labels
1.7	Food preparation	Factors associated with how food is prepared for consumption (Larson et al., 2006).
2.1	Intentions	What one intends or plans to choose, buy, or consume (Conner et al., 2002).
2.2	Eating habits	Umbrella term for outcomes related to the actual act of consumption.
2.3	Eating occasions	The typical/habitual eating behaviors that one has developed over time, often triggered automatically in response to contextual cues that have been associated with their performance (Gardner, 2015).
2.4	Frequency	How often one eats (Gomez et al., 2015).
2.5	Time	When one eats (which moment of the day) (Kupek et al., 2016).
2.6	Rate of eating	The speed at which one eats a certain amount of food.
2.7.1	Portions size	A specification of the amount one eats in terms of the size of a standard portion (Nielsen and Popkin, 2003).
2.7.2	Portion number	A specification of the amount one eats in terms of the amount of standard portions (Benton, 2015).
2.8	Food and drink substitution	The exchange of one food or drink item for another (commonly the substitution of an unhealthy food for a healthier one)
2.9	Disordered eating symptoms	Signals of abnormal eating (e.g., anorexia and binge eating) (Rohde et al., 2015).
2.10	Neophobia/pickiness/ fussiness	Different aspects of selective eating (Dovey et al., 2008).
3.1.1	Type of pattern	Characterization of the dietary pattern, e.g., in Western, Mediterranean, and Prudent (Hu, 2002).
3.1.2	Diversity of pattern	Diversity of foods and beverages consumed (within a specific pattern) (Hu, 2002).
3.1.3.1	Healthiness- unhealthy intake	Consumption of (combinations) of foods and beverages attributed to have a negative effect on one's health, e.g., sugar sweetened beverages (Ocké, 2013).
3.1.3.2	Healthiness- healthy intake	Consumption of (combinations) of foods and beverages attributed to have a positive effect on one's health, e.g., fruits and vegetables (Ocké, 2013).
3.2.1	Meal content	Combination of food items within a meal (Leech et al., 2015b).
3.2.2	Caloric and nutrient intake distribution	Distribution of (daily) energy and nutrient intake across the main meals and snacks (Leech et al., 2015b).
3.3	Total energy intake	Total amount of energy consumed within a specific time frame (mostly per day)
3.4	3.4 Food and non-alcoholic drink intake	Amount/servings of food items one eats within a specific time frame (mostly per day) (Illner et al., 2010).
3.5.1	Food and non-alcoholic drink components- nutrients	The nutrients (carbohydrates, protein, fat, vitamins, minerals, and water) in a food item (Coultate, 2009).

3.5.2	Food and non-alcoholic drink components- energy	The caloric content of a food item (Coultate, 2009).
3.5.3	Food and non-alcoholic drink components- other	Non-nutritive food components of a food item (Coultate, 2009).
3.6	Alcoholic drink intake	The consumption of alcoholic beverages.
