# Supplementary material for: Identifying and measuring the behavioural, dietary, and physical activity components of weight management consultations delivered by general practice nurses in routine care
Source: BMC Fam Pract. 2021 Apr 7;22:65. doi: 10.1186/s12875-021-01403-1 (PMC8028812; doi:10.1186/s12875-021-01403-1)
Supplement: Supplementary file 3 — Additional file 3. Coding rulebook. [file 12875_2021_1403_MOESM3_ESM.pdf]

Additional file 3

Coding rulebook

General

How to code:

0- BCT not used
1- BCT present in all probability
2- BCT present beyond all reasonable doubt

We are coding what the nurse is saying to the patient. Not sufficient for the nurse to say 'yes' or 'no' in agreement to suggestions from the participant. However, it is sufficient to code if the nurse continues a discussion with the participant about the recommendation for an elongated period of time.

Only code the overarching meaning of the sentence.
E.g. when the nurse is discussing portion sizes of a specific food, code about portion size not the specific food.

No benefit of doubt, just use what has been actually said in the appointment.

Regarding the nurse questioning the participant**:**
Only code this if the nurse is asking questions that are suggestive of the future or intended to encourage future behaviour. Do not code if the nurse is asking about past behaviour- dietary, behavioural or physical activity assessment is not sufficient to code.
E.g. If nurse suggests to participant they try a particular activity- 'have you considered trying swimming?' this is sufficient to code 1 (but not 2) for structured activity.
If the nurse asks the participant 'Have you swam before?' this isn't sufficient to code.

BCT

Specific BCTs

(BCT/ definition/ how to code / example)

1.1 Goal setting (behaviour)- 'set or agree on a goal defined in terms of the behaviour to be achieved. Note: only code goal-setting if there is sufficient evidence that goal set as part of intervention.'
Only code goal setting in the context where the overarching theme is to set a goal. For example, if the nurse mentions to the patient that they should eat 1500kcal/day and clearly sets this as a goal or gets the patient to agree that this is a goal, then it is coded. If the nurse were to mention the patient should eat 1500kcal whilst in a list of discussing other matters or it is clear the possible introduction of setting a goal is not the overarching part of the conversation, do not code as this is not sufficient.

1.5 Review of behaviour goals- 'review behaviour goal(s) jointly with the person and consider modifying goal(s) or behaviour change strategy in light of achievement. This may lead to re-setting the same goal, a small change in that goal or setting a new goal instead of (or in addition to) the first, or no change.
It is not sufficient to code if the nurse asks about the goal only. They must also discuss the future of the goal (whether that be changing the behaviour goal or continuing as is). There must be a 2 way dialogue with the patient so we can see patient engagement with the goal
E.g. We would not code when the nurse asks how portion sizing reduction is going, but would code

this if the nurse also suggested that she either continued to reduce portion sizes or gave her a new goal such as reducing the amount of sugary drinks she ate too.

2.2 Feedback on behaviour- ‘monitor and provide informative or evaluative feedback on performance of the behaviour (e.g. form, frequency, duration, intensity).
Code 2 if the nurse monitors AND provides feedback. If provides feedback after participant discusses their behaviour, code 1.
E.g. patient discusses about eating mayonnaise (behaviour) and nurse acknowledges then provides feedback about behaviour.

4.1 Instruction on how to perform a behaviour- ‘advise or agree on how to perform the behaviour’. Includes changing how a behaviour is carried out, not just how to perform a behaviour.
E.g. nurse tells patient they need to walk faster so heart beats faster.

5.1 Information about health consequences- ‘provide information (e.g. written, verbal, visual) about health consequences of performing the behaviour’
It is not sufficient to code this unless a disease is presented. Do not code if information about symptoms. Do code if the nurse explains weight loss will be good for your health- 1. If nurse says losing X amount of weight will benefit your health code 2. Also consider whether this is in the context of goal setting.
E.g. do not code that apple will fill you up. Do not code that exercise helps with stress

5.5 Anticipated regret- ‘induce or raise awareness of expectations of future regret about performance of the unwanted behaviour’
Code if the overarching theme of the conversation is of awareness of the regret. Don’t code this when the nurse is discussing how to deal with the consequences.
E.g. code if the nurse tells the patient they will feel guilty about eat a whole tub of ice cream. Don’t code if the nurse tells the patient they should feel okay about failing the diet. This is because they are informing how to deal with the consequence, not what the consequence will be.

8.2 Behavioural substitution- ‘prompt substitution of the unwanted behaviour with a wanted or neutral behaviour’.
We code this when in the context of swapping food choice.
E.g. the nurse suggests the participant eats a low calorie rich tea biscuit instead of an energy dense one.

9.1 Credible source- ‘Present verbal or visual communication from a credible source in favour of or against the behaviour’ Note: code this BCT if source generally agreed on as credible e.g. health professionals, celebrities or words used to indicate expertise or leader in field and if the communication has the aim of persuading.
Key to note that the source is being used to persuade- look at the overarching message of what the nurse has said and if it seems they are using credible source for a purpose rather than just mentioning as part of a sentence, code.
E.g. Code the use of the BHF document if the nurse states that they are using a BHF document. Don’t code if the nurse uses the BHF document but doesn’t say that she is using it.

10.4 Social reward ‘Arrange verbal or non-verbal reward if and only if there has been effort and/ or progress in performing the behaviour (includes positive reinforcement)’.
Code 1 if the nurse gives general social reward i.e. not about a specific behaviour, action or behaviour outcome the participant has carried out.
Code 2 if the nurse gives reward about a specific behaviour, action or outcome behaviour

E.g. at the end of a consultation a nurse congratulates and says well done, but not to anything in particular but generally just from the outcome of the appointment, code 1. If the nurse congratulates the participant for losing 1kg after weighing them this would be coded as 2.

13.2 Framing/ reframing- 'suggest the deliberate adoption of a perspective or new perspective on behaviour (e.g. its purpose) in order to change cognitions or emotions about performing the behaviour.
Code 1 if the nurse suggests a change in belief without the patient having previously stated this belief, code 2 if belief is stated and then nurse tried to change
E.g. code 1 if the nurse says this is a lifestyle, not a diet
Code 2 if the patient believes that they will gain 2 stone from giving up smoking and the nurse tells them this is wrong and they should expect no more of a weight gain than a few kgs.

Specific examples

Cutting down/ reducing a specific food- e.g. have half a potato instead of a full potato.
Code 1.2 problem solving, not 8.2 behavioural substitution.
If food were changed, e.g. instead of cake eat an apple this would be 8.2

Diet and exercise

1.2 Ability to pay- 'the extent to which one is able to buy certain food products'.
The nurse can guide the patient as to where or how they can buy products to help aide weight loss. E.g. 'most supermarkets now have good prices on fruit and vegetables' or the nurse gives the participant a free gym membership.

1.6 Product of purchase- 'which food product is purchased'
Code 1 when discussing using food labels to help choice of food.

2.1 Intentions- 'What one intends or plans to choose, buy or consume'
Code when the nurse encourages the patient to think about/ change their intentions. Code 2 in the context of when the nurse suggests to the patient they should plan their meals.

2.3 Eating occasions- 'The actual eating event, may include also the physical and social context in which one eats (e.g. how often and when one eats at home; which portion size or number one eats when being with others)'
Code when talking about eating in a specific social or physical environment.
E.g. code when discussing about eating cake at a birthday party (if related to what we are looking at i.e. if nurse uses as a method to help patient lose weight).
E.g. code when nurse and patient discuss plate sizes- having a smaller plate size can lead to a reduction in portion size.

2.5 Time- 'When one eats (which moment of the day)'
Code when the nurse encourages the patient to eat meals at certain times of the day- we can assume that breakfast is in the morning, lunch around midday and dinner in the evening so these fall under time.
E.g. the nurse recommends the patient to start eating breakfast would be coded as 2.

2.6 Rate of eating- 'The speed at which one eats a certain amount of food.'
This would be coded if the nurse suggests the participant should consider changing the speed at which they eat (this may be because slowing the rate of eating allows patients to become more aware of when they feel 'full up')

E.g. if the nurse tells the patient to eat their sandwich more slowly or encourage the patient to be the last person to finish eating in a social context

2.7 Portion size/ number
If the nurse discusses overall reduction of portions over a day, code portion size
E.g. If you put in too much food throughout the day you won't lose weight

2.8 Food and drink substitution- 'The exchange of one food or drink item for another (commonly the substitution of an unhealthy food for a healthier one)'
E.g. 'Low fat yoghurts may be very high in sugar, so try to select natural yoghurts instead and add some fruit or nuts'

3.1.1 Type of pattern- 'Characterisation of the dietary pattern e.g. in Western, Mediterranean and Prudent'
Code when discussing the eat well plate, rather than 3.2.1 meal content (unless nurse is very clearly discussing a meal)

3.1.2 Diversity of pattern- 'diversity of food and beverages consumed (within a specific pattern)'
Commonly, nurses encourage patients to not completely give up eating large food groups, or specific foods. This is coded under diversity of pattern. Code 2 when nurse states that nothing should be denied from patients diet.
The eatwell plate may also fall into this category if discussed in the context of diversity of food groups.
E.g. 'If you cut out all foods that you enjoy you will struggle with dieting' or 'Don't cut out whole food groups because you may not receive all the vitamins and minerals required for a healthy diet'.

3.1.3.1/ 3.1.3.2 Healthiness/ unhealthy intake- 'Consumption of (combinations) of foods and beverages attributed to have a negative effect on one's health e.g. sugar sweetened beverages.
Code this only when health implications are discussed. It is not sufficient to code this if the unhealthy part of the food item is mentioned
Also coded it is specified explicitly that this is a healthy food
E.g. You would code that fruit juices contain a lot of sugar which can be bad for your teeth and blood sugar levels.
You wouldn't code that crisps are high fat. This would go under the section 3.4.1 Food and drink components- nutrients.
E.g. Code 'apples are healthy so you should eat them' or 'chocolate is high in fat and so unhealthy'

3.2.2 Caloric and nutrient intake distribution- 'Distribution of (daily) energy and nutrient intake across the main meals and snacks'
Code if the nurse has stated the total calories/ nutrients and then allocates these to either a specific meal or snacks
e.g. you have 200 calories allocated to snacks would be coded.

3.3 Total energy intake
Code 1 if mentions caloric intake without specific values
Code 2 if specific values are used e.g. eat 1500kcal/day

3.6 Alcoholic drink intake- 'consumption of alcoholic beverages'
Code when the nurse discusses alcoholic consumption (note point 'regarding the nurse questioning the participant')
E.g. A small glass of wine contains 100 calories so you should limit the amount you drink.

Specific examples

When a nurse mentions that the patient should generally exercise more, code this as ‘lifestyle type other’.
