# Supplementary material for: Identifying and measuring the behavioural, dietary, and physical activity components of weight management consultations delivered by general practice nurses in routine care
Source: BMC Fam Pract. 2021 Apr 7;22:65. doi: 10.1186/s12875-021-01403-1 (PMC8028812; doi:10.1186/s12875-021-01403-1)
Supplement: Supplementary file 4 — Additional file 4. Physical activity recommendations and their definitions used by coders. [file 12875_2021_1403_MOESM4_ESM.pdf]

Additional file 4. Physical activity recommendations and their definitions used by coders.

Reference	Name	Definition
1	Sedentary time	Being sedentary.
2.1	Lifestyle activity type walking	Walking as a form of physical activity.
2.2	Lifestyle activity type other	A lifestyle activity that is not walking as a form of physical activity.
2.3	Frequency	Frequency of a lifestyle activity, walking or other.
2.4	Intensity	Intensity of a lifestyle activity, walking or other.
2.5	Duration	Duration of a lifestyle activity, walking or other.
3.1	Structured activity type e.g. sports	A structured activity that is not a lifestyle activity, frequently a sport or going to the gym.
3.2	Frequency	Frequency of a structured activity.
3.3	Intensity	Intensity of a structured activity.
3.4	Duration	Duration of a structured activity.
