# Supplementary material for: Identifying and measuring the behavioural, dietary, and physical activity components of weight management consultations delivered by general practice nurses in routine care
Source: BMC Fam Pract. 2021 Apr 7;22:65. doi: 10.1186/s12875-021-01403-1 (PMC8028812; doi:10.1186/s12875-021-01403-1)
Supplement: Supplementary file 5 — Additional file 5. Supplementary results. [file 12875_2021_1403_MOESM5_ESM.pdf]

Additional file 5

**Table S1. Correlation coefficients (p-value) between the average number of BCTs or recommendations used per consultation per participant and percentage weight change at 3, 6 and 12 months.**

| | Average number of BCTs per consultation per participant | Average number of dietary recommendations per consultation per participant | Average number of physical activity recommendations per consultation per participant |
|---|---|---|---|
| 3-month percentage weight change (n=40) | -0.01 (0.94) | 0.00 (1.00) | 0.29 (0.07) |
| 6-month percentage weight change (n=39) | -0.02 (0.89) | -0.21 (0.21) | 0.24 (0.15) |
| 12-month percentage weight change (n=40) | 0.23 (0.16) | 0.04 (0.83) | 0.36 (0.02) |

N= number of participants

**Table S2. Comparisons of the number of BCTs, dietary and physical activity recommendations per consultations of participants that lost < andlost ≥5% of their baseline body weight at 6 months (mean ± standard deviation) with p-values of independent T-tests.**

| | **Participants with <5% weight loss at 6 months n=20** | **Participants with ≥ 5% weight loss at 6 months n=19** | **Independent T-test p-value** |
|---|---|---|---|
| Mean number of BCTs coded as present per consultation | 3.5 (SD 1.2) | 3.8 (SD 1.3) | 0.52 |
| Mean number of dietary recommendations coded as present per consultation | 3.2 (SD 2.5) | 3.1 (SD 2.3) | 0.56 |
| Mean number of physical activity recommendations coded as present per consultation | 1.4 (SD 1.2) | 0.9 (SD 0.9) | 0.26 |

**Table S3. Comparison of BCT, dietary recommendations and physical activity recommendations used in consultations among participants that lost < and lost ≥5% baseline weight at 6 months with Chi-squared p-values and Fisher's exact test where appropriate.**

| | % used in consultations | | |
|---|---|---|---|
| **BCT or recommendation** | **Of the 20 participants with <5% weight loss at 6 months n=62** | **Of the 19 participants with ≥5% weight loss at 6 months n=59** | **Chi squared p-value, *Fisher's exact test** |
| ***BCT*** | | | |
| Feedback on outcome(s) of behaviour | 77.4 | 79.7 | 0.76 |

| | | | |
|---|---|---|---|
| Problem solving | 43.6 | 37.3 | 0.48 |
| Social reward | 29.0 | 52.5 | 0.01 |
| Instruction on how to perform a behaviour | 29.0 | 20.3 | 0.27 |
| Self-monitoring of behaviour | 24.2 | 27.1 | 0.71 |
| Biofeedback | 21.0 | 23.7 | 0.72 |
| Framing/ reframing | 25.8 | 33.9 | 0.33 |
| Information about health consequences | 19.4 | 15.3 | 0.55 |
| Social comparison | 16.1 | 10.2 | 0.33 |
| Self-monitoring of outcome(s) of behaviour | 8.1 | 8.5 | 0.93 |
| Credible source | 4.8 | 13.6 | *0.12 |
| Feedback on behaviour | 4.8 | 10.2 | *0.32 |
| Behaviour substitution | 11.3 | 3.4 | *0.16 |
| Restructuring the physical environment | 9.7 | 3.4 | *0.27 |
| Graded tasks | 3.2 | 8.5 | *0.26 |
| Goal setting (behaviour) | 3.2 | 5.1 | *0.67 |
| Monitoring outcome(s) of behaviour by others without feedback | 6.5 | 1.7 | *0.37 |
| Monitoring of emotional consequences | 0.0 | 6.8 | *0.11 |
| Goal setting (outcome) | 4.8 | 1.7 | *0.62 |
| Non-specific incentive | 3.2 | 1.7 | *1.00 |
| Review behaviour goal(s) | 0.0 | 3.4 | *0.24 |
| Social support (unspecified) | 1.6 | 1.7 | *1.00 |
| Reduce negative emotions | 0.0 | 1.7 | *0.49 |
| Body changes | 0.0 | 1.7 | *0.49 |
| Social support (practical) | 0.0 | 1.7 | *0.49 |
| Pharmacological support | 1.6 | 0.0 | *1.00 |
| Distraction | 1.6 | 0.0 | *1.00 |
| Adding objects to the environment | 1.6 | 0.0 | *1.00 |
| ***Dietary recommendations*** | | | |
| Portions size | 40.3 | 33.9 | 0.47 |
| Food and non-alcoholic drink components- nutrients | 30.7 | 28.8 | 0.83 |
| Diversity of pattern | 27.4 | 25.4 | 0.80 |
| Food and non-alcoholic drink components- energy | 19.4 | 22.0 | 0.72 |
| Total energy intake | 14.5 | 15.3 | 0.91 |
| Portion number | 21.0 | 13.6 | 0.28 |
| Food and drink substitution | 16.1 | 15.3 | 0.90 |

| | | | |
|---|---|---|---|
| Food preparation | 14.5 | 18.6 | 0.54 |
| Caloric and nutrient intake distribution | 19.4 | 13.6 | 0.39 |
| Frequency | 17.7 | 13.6 | 0.53 |
| Food and non-alcoholic drink intake | 16.1 | 15.3 | 0.71 |
| Type of pattern | 16.1 | 13.6 | 0.69 |
| Alcoholic drink intake | 11.3 | 13.6 | 0.90 |
| Product of purchase | 12.9 | 10.2 | 0.64 |
| Eating occasions | 8.1 | 10.2 | 0.69 |
| Meal content | 6.5 | 8.5 | *0.74 |
| Healthiness- healthy intake | 8.1 | 1.7 | *0.21 |
| Time | 6.5 | 1.7 | *0.37 |
| Food and non-alcoholic drink components- other | 1.6 | 5.1 | *0.36 |
| Intentions | 3.2 | 3.4 | *1.00 |
| Eating habits | 3.2 | 3.4 | *1.00 |
| Rate of eating | 1.6 | 1.7 | *1.00 |
| Willingness to pay | 0.0 | 1.7 | *0.49 |
| Ability to pay | 0.0 | 1.7 | *0.49 |
| Preferences | 0.0 | 1.7 | *0.49 |
| ***Physical activity recommendations*** | | | |
| Lifestyle activity type walking | 32.3 | 32.2 | 1.00 |
| Structured activity type e.g. sports | 27.4 | 23.7 | 0.64 |
| Intensity (of lifestyle activity) | 22.6 | 13.6 | 0.20 |
| Lifestyle activity type other | 17.7 | 8.5 | 0.13 |
| Duration (of lifestyle activity) | 11.2 | 6.8 | *0.53 |
| Frequency (of structured activity) | 11.2 | 1.7 | *0.06 |
| Intensity (of structured activity) | 11.2 | 1.7 | *0.06 |
| Duration (of structured activity) | 9.7 | 5.1 | *0.49 |
| Frequency (of lifestyle activity) | 8.1 | 1.7 | *0.21 |

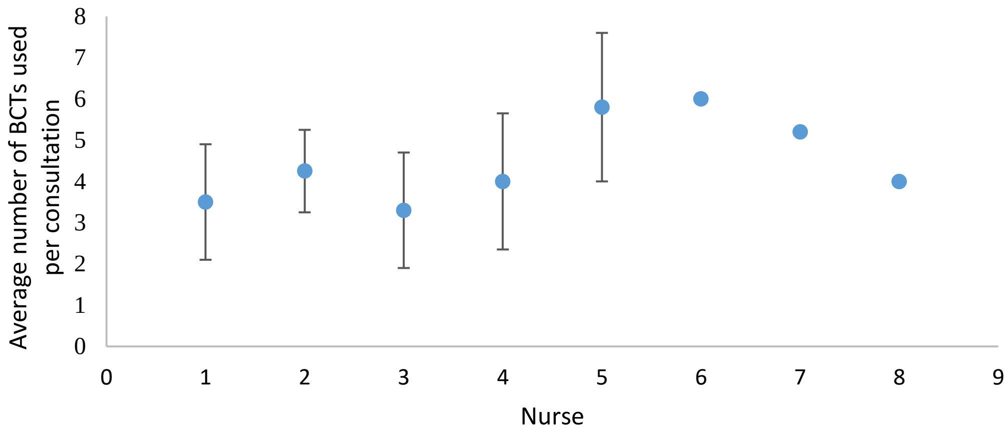


**Figure S1. BCT usage (mean ±standard deviations) for individual nurses.**

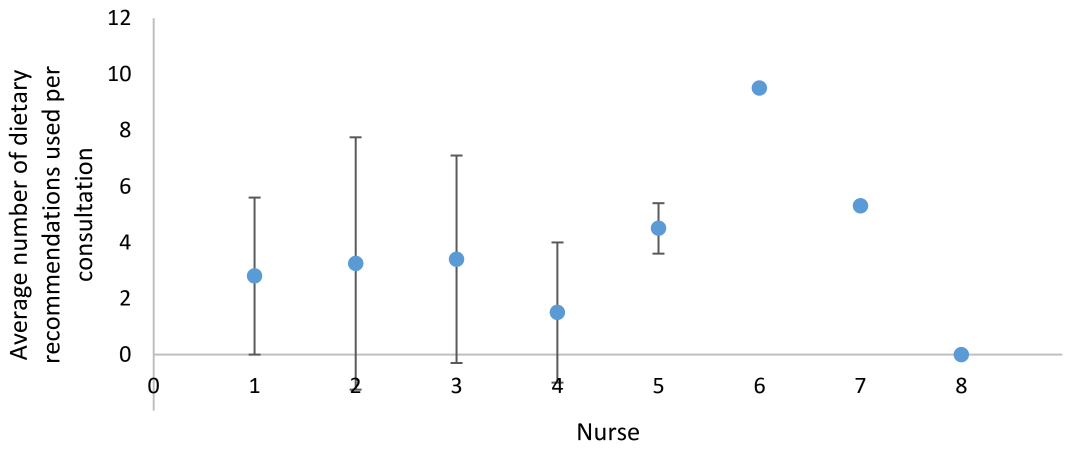


**Figure S2. Dietary recommendation usage (mean ±standard deviations) for individual nurses.**

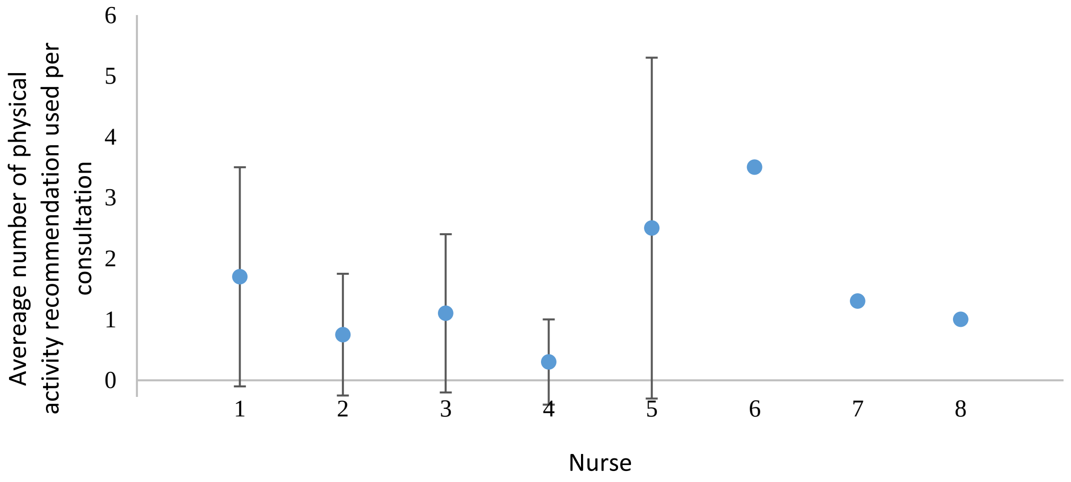


**Figure S3. PA recommendation usage (mean ±standard deviations) for individual nurses.**

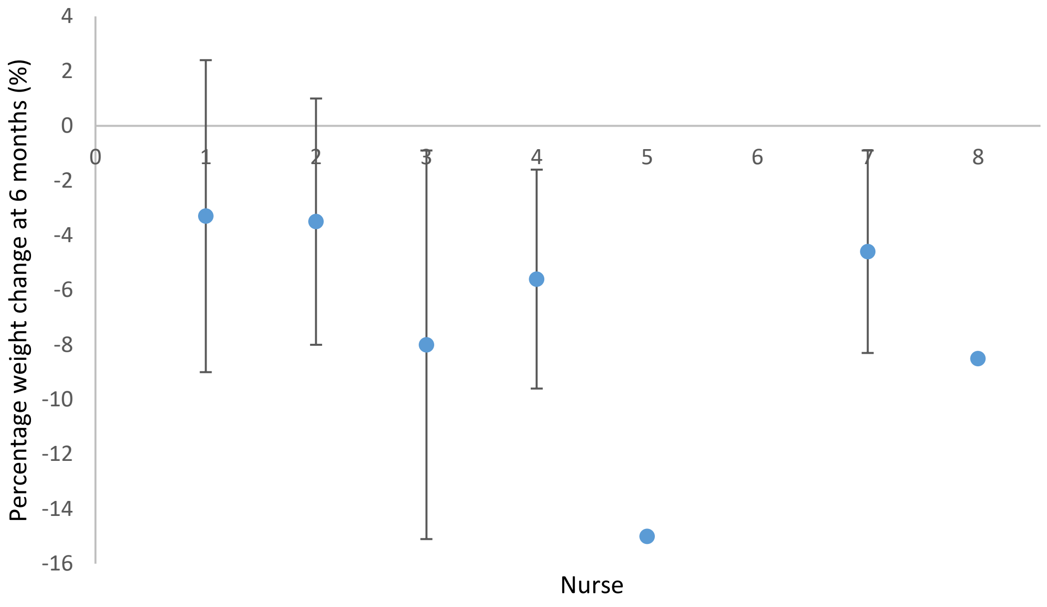


**Figure S4. Percentage weight change (mean ±standard deviation) of individual nurses' groups of participants.**
